# Genomic Surveillance of Respiratory Syncytial Virus in Sydney Reveals Rebound in Postpandemic Viral Diversity

**DOI:** 10.1093/ofid/ofag192

**Published:** 2026-04-02

**Authors:** Julia M S Herkes, Charles S P Foster, Alice Michie, Adam W Bartlett, William D Rawlinson, Gregory J Walker

**Affiliations:** Virology Research Laboratory, Serology and Virology Division (SAViD), NSW Health Pathology, Prince of Wales Hospital, Randwick, New South Wales, Australia; School of Clinical Medicine, Faculty of Medicine, University of New South Wales, Kensington, New South Wales, Australia; Virology Research Laboratory, Serology and Virology Division (SAViD), NSW Health Pathology, Prince of Wales Hospital, Randwick, New South Wales, Australia; School of Biomedical Sciences, Faculty of Medicine, University of New South Wales, Kensington, New South Wales, Australia; Virology Research Laboratory, Serology and Virology Division (SAViD), NSW Health Pathology, Prince of Wales Hospital, Randwick, New South Wales, Australia; Infectious Diseases, Sydney Children's Hospital, Randwick, New South Wales, Australia; Virology Research Laboratory, Serology and Virology Division (SAViD), NSW Health Pathology, Prince of Wales Hospital, Randwick, New South Wales, Australia; School of Clinical Medicine, Faculty of Medicine, University of New South Wales, Kensington, New South Wales, Australia; School of Biomedical Sciences, Faculty of Medicine, University of New South Wales, Kensington, New South Wales, Australia; Virology Research Laboratory, Serology and Virology Division (SAViD), NSW Health Pathology, Prince of Wales Hospital, Randwick, New South Wales, Australia; School of Biomedical Sciences, Faculty of Medicine, University of New South Wales, Kensington, New South Wales, Australia

**Keywords:** fusion protein, genomic diversity, mutation, respiratory syncytial virus, therapeutic resistance

## Abstract

Respiratory syncytial virus (RSV) transmission between 2020 and 2025 was impacted by COVID-19-related public health measures, resulting in unprecedented genomic bottlenecks, and postpandemic surges in clinical cases. We performed whole-genome sequencing of 100 infant RSV cases in Sydney during the 2024 winter season. Phylogenetic analysis revealed a marked rebound in genomic diversity compared with prior seasons, driven by multiple international lineage introductions. No known therapeutic-resistance mutations were detected. These findings provide a preintervention baseline for monitoring RSV evolution under emerging therapeutic selection pressures in Australia, with the national rollout of the recently approved maternal vaccine (Abrysvo) and monoclonal antibody (nirsevimab) through the RSV Mother and Infant Protection Program commencing in 2025.

## Teaser

Genomic surveillance of respiratory syncytial virus in Sydney during 2024 revealed a rebound in postpandemic diversity driven by multiple international introductions, establishing a baseline for monitoring viral evolution as prophylactic therapeutics are made nationally available.

Respiratory syncytial virus (RSV) is the most common causative agent of acute respiratory tract infections among Australian children under 4 years old, accounting for more laboratory-confirmed detections per year than influenza and severe acute respiratory syndrome coronavirus 2 (SARS-CoV-2) combined [1]. Whilst most patients experience self-limiting, cold-like symptoms, neonates and infants less than 6 months of age are at substantially greater risk of developing severe lower respiratory tract infections (LRTIs), even in the absence of medical comorbidities [2, 3]. Infection with RSV during this critical period of lung and immune system development may give rise to self-perpetuating cycles of respiratory sequelae, including persistent wheezing, recurrent all-cause LRTIs, asthma, and reduced lung function [4]. Infection does not confer long-lasting immunity, as neutralizing antibodies rapidly wane to preinfection titer [5]. Consequently, reinfection can occur throughout life, particularly during annual seasonal epidemics that typically span from autumn to early spring in the temperate regions of Australia [6].

The transmission and seasonality of RSV was profoundly disrupted by public health interventions introduced in response to the coronavirus disease 2019 (COVID-19) pandemic. Most notably, there was a near-absence of RSV cases in Australia during the expected winter season peak between March and September, leading to a collapse in genomic diversity [6, 7]. The easing of COVID-19-related restrictions in late 2020 to early 2021 coincided with atypical off-season epidemics of 2 geographically distinct RSV-A lineages on the east and west coasts of Australia [7], mirroring genomic bottlenecks reported in New Zealand, Europe, and in some regions of USA [8]. While the seasonality of RSV returned to prepandemic trends in Australia by winter of 2021, reported case numbers surged approximately 3-fold in 2022 in comparison with prepandemic years, as international travel restrictions were removed [6]. Genomic surveillance of RSV in Sydney during this period revealed that this resurgence in cases was comprised of small number of preexisting viral lineages [2].

The need for ongoing genomic surveillance of RSV has become increasingly important following the recent approval of prophylactic therapeutics. These include a maternal vaccine (Abrysvo) and monoclonal antibody (nirsevimab) for infants, as well as several vaccines for older adults. Widespread use of these therapeutics may exert selective pressure on the virus, particularly the targeted fusion (F) protein, potentially driving the emergence of escape variants. Such variants have already been detected in Europe at low frequencies, where the rollout of nirsevimab preceded that in Australia [9]. There is a need to establish a preintervention baseline of RSV genomic diversity in Australia, particularly among infants, to serve as a comparator as the national RSV Mother and Infant Protection Program commences in 2025. Here, we describe the genomic diversity of RSV circulating among infants in Sydney, Australia during 2024 through whole-genome sequencing of clinical samples. We also characterize mutations within the *F* gene to identify viral variants with the potential to confer resistance to established and novel prophylactic therapeutics palivizumab, nirsevimab, and Abrysvo.

## MATERIALS AND METHODS

### Sample Collection

Nasopharyngeal swabs (NPS) were collected for routine diagnostic testing in children who presented to Sydney Children's Hospital (SCH), Randwick, Australia, with respiratory symptoms. Respiratory syncytial virus infection and subtype were determined at the time of presentation by the Serology and Virology Division, New South Wales Health Pathology, using the Allplex Respiratory Panel 1 (Seegene, South Korea) protocol. Immediately following diagnostic reverse transcription real-time polymerase chain reaction (RT-qPCR), RSV-positive NPS with > 500 µL remnant volume were frozen at −80°C until transport to the Virology Research Laboratory for genomic sequencing.

Selection criteria for inclusion in this study were NPS from RSV-positive infants < 12 months of age at time of presentation between March and August 2024, with diagnostic RT-qPCR cycle threshold (Ct) value ≤ 31. A total of 100 NPS were eligible for inclusion in this study.

### Sample Preparation and Whole Genome Sequencing

Total nucleic acid was extracted from 200 µL of remnant NPS using the MagNA Pure 96 DNA and Viral NA Small Vol Kit (Roche Diagnostics, Australia) as per the manufacturer's protocol and eluted at a volume of 50 µL. cDNA was synthesized as per the SuperScript IV First-strand cDNA Synthesis Kit (Thermo Fisher Scientific, Lithuania) protocol. Tiled RSV-A and RSV-B amplicons were generated from cDNA using the ARTIC RSV Primer Scheme protocol, described by Maloney et al [10], with the Q5 High-Fidelity 2X Mastermix (Biolabs, USA). Cleanup was performed using DNA purification beads (Twist Bioscience, USA) as per the manufacturer's protocol. The dsDNA concentration (ng/µL) was quantified using the PicoGreen dsDNA Kit (Thermo Fisher Scientific, USA) as per the manufacturer's protocol.

RSV amplicons were prepared for WGS using the Illumina DNA Library Prep Kit (Illumina, USA) as per the manufacturer's protocol, with a DNA input of 100–500 ng per sample. Libraries were sequenced in-house on an Illumina MiSeq (paired-end, 2 × 150 bp), using the MiSeq Reagent Kit v2 (Illumina, USA).

### Bioinformatic Analysis

Bioinformatic analysis of raw sequencing reads was achieved using a custom in-house RSV bioinformatics pipeline [11]. Raw sequencing reads were trimmed and quality filtered using fastp (v0.23.4) [12] with automatic detection of residual sequencing adapters, error correction, a requirement of a mean quality threshold of Q20 in sliding windows from the start and end of reads, and a minimum read length of 50 nt. Filtered reads were then mapped to the hRSV/A/England/397/2017 (EPI_ISL_412866) and hRSV/B/Australia/VIC-RCH056/2019 (EPI_ISL_1653999) reference genomes using bwa-mem (v0.7.19) [13]. These genomes possess the G-gene duplications (RSV-A: 72 nt; RSV-B: 60 nt) that are characteristic of currently circulating RSV-A and RSV-B strains. Variants were called against these reference genomes using LoFreq (v2.1.5) [14] and filtered by sequencing depth (minimum 10 reads), allele frequency (minimum 0.03) and quality threshold (minimum 20) with bcftools (v1.21) [15]. The amino acid consequences of filtered variants with an allele frequency > 0.5 were also inferred using bcftools csq. Consensus genomes were assembled with bcftools, incorporating variants with a minimum depth of 10 reads. Finally, lineages were assigned using Nextclade (v3.17.0) [16].

For phylogenetic analysis, 481 whole RSV genomes (>14 900 nt) sampled globally between 2020–2024 were downloaded from the GISAID and Sequence Read Archive databases (Supplementary Appendix Table 1) [17]. These included 229 sequences previously generated by Walker et al [2] from infants presenting to SCH, Randwick, Australia, during a 2022 surge in RSV cases. Five historic RSV-A and RSV-B sequences were downloaded from the GISAID and NCBI databases, respectively, to use as outgroup taxa (Supplementary Appendix Table 2) [18]. A multiple sequence alignment was generated using these downloaded sequences and the 100 novel sequences generated in this study using MAFFT (v7), followed by manual adjustments [19]. Maximum-likelihood phylogenetic trees were inferred using IQ-TREE (v2) [20], with the best-fit nucleotide substitution model (GTR + F + I + G4) estimated by the ModelFinder function and support estimated via 1000 ultrafast bootstrap replicates and 1000 Shimodaira-Hasegawa approximate likelihood ratio test replicates [21–23].

### Data Processing and Visualization

Outputs of the bioinformatics pipeline, including sequencing metrics, lineage assignment, and variant data were summarized using R (v2024.12.1 + 563) (Supplementary Appendix Table 3) [24]. Phylogenetic trees were plotted and annotated according to compiled metadata in R, using the tidyverse, treedataverse, ape, ggtree, and phylobase packages [25–29].

### Ethics Approval

This study was approved by the Sydney Children's Hospitals Network Human Research Ethics Committee (2020/ETH00718). The requirement for informed consent was waived by the committee, as the study was non-interventional and used retrospective de-identified residual specimens for virological analysis.

## RESULTS

### Sample Characteristics and Sequencing Metrics

Whole RSV genomes were generated from 100 RSV-positive infants who presented to SCH, Randwick, Australia, between March and August 2024, corresponding with the peak in laboratory-confirmed RSV detection [6]. More RSV-A (n = 68) samples were eligible for inclusion than RSV-B (n = 32), with no mixed infections identified (Supplementary Appendix Table 4). The median RT-qPCR Ct value was 20.76 (IQR: 18.97–23.98) (Supplementary Appendix Table 4).

The median genome coverage was 98.5% [IQR: 98.0%–99.0%] at a mean depth of 999.7 reads per sample (SD = 387.9). Whole RSV genome sequences, defined as > 95% coverage, were obtained from 93 of 100 samples. Coverage of the remaining 7 samples ranged between 87.2% and 94.7%.

### RSV Lineages

Lineages were assigned based on the whole or near full-length genomes generated as per the 2024 Goya classification [30]. All RSV-A samples belonged to 6 distinct lineages of the A.D G-clade (Table 1). Of these, A.D.1.4 (n = 35, 51%) and A.D.1.5 (n = 16, 24%) were the most common lineages. All RSV-B samples belonged to 5 lineages of the B.D G-clade (Table 1). The majority of RSV-B samples belonged to lineage B.D.E.1 (n = 21, 66%).

**Table 1.** **Frequency and Relative Frequency (%)** ^a^  **of RSV Lineages Circulating Among 100 Infected Infants < 12 Months of Age Who Presented to Sydney Children's Hospital, Randwick, Australia Between March and August 2024, by Subtype**

Subtype	Lineage^b^	No. (%).
RSV-A (n = 68)	A.D.1.4	35 (51)
	A.D.1.5	16 (24)
	A.D.1.6	1 (1)
	A.D.3	5 (7)
	A.D.5.1	3 (4)
	A.D.5.2	8 (12)
RSV-B (n = 32)	B.D.4.1.1	1 (3)
	B.D.E.1	21 (66)
	B.D.E.1.2	6 (19)
	B.D.E.1.8	1 (3)
	B.D.E.5	3 (9)

^a^Relative frequency of each lineage is given as a percentage of total samples from the respective subtype.

^b^Lineages were assigned using Nextclade (v3.17.0) by aligning consensus genomes to the Nextclade dataset (2025–09–09).

### Phylogenetic Analysis

**Figure 1.**
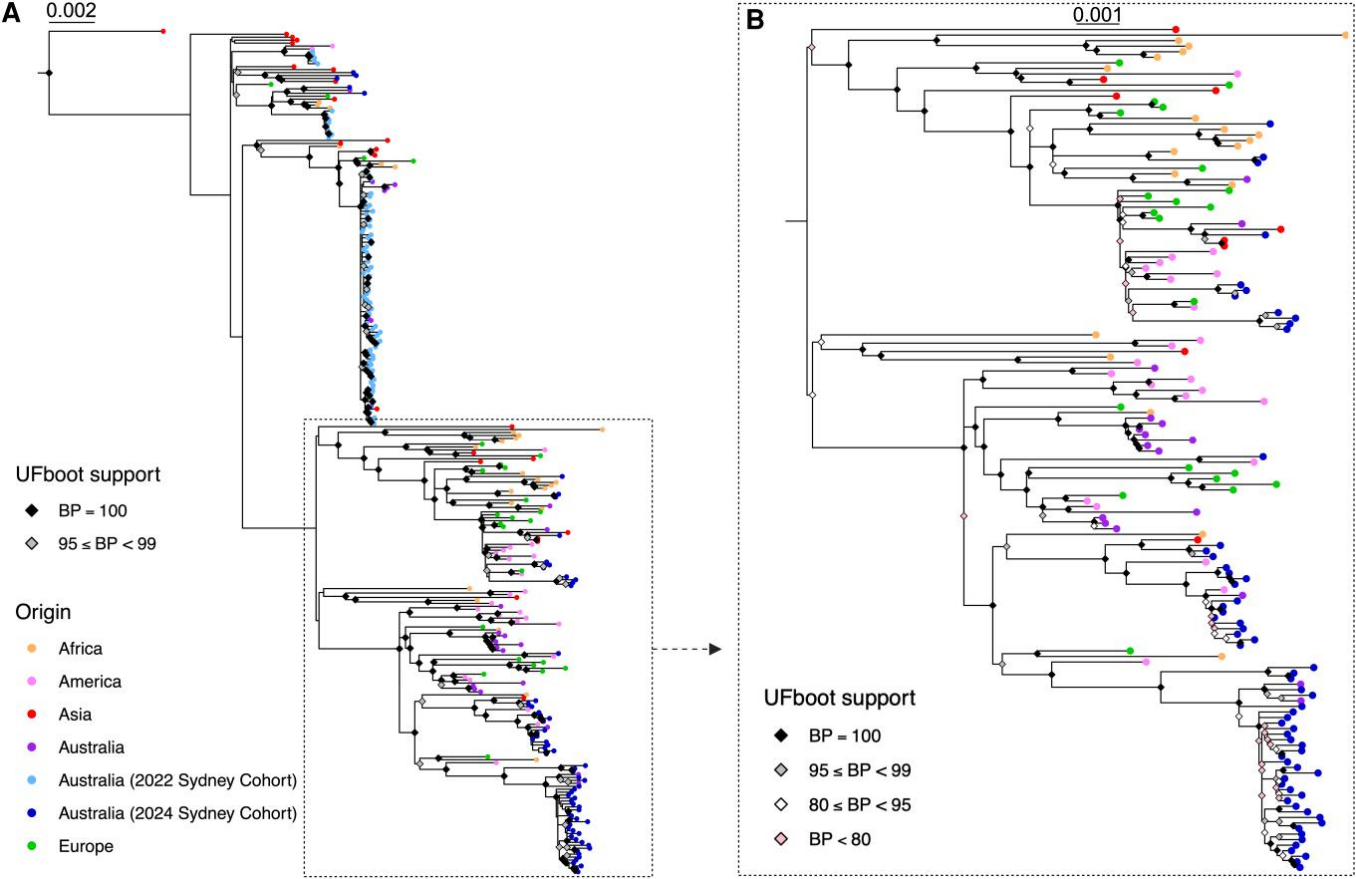
Phylogeny of RSV-A. *A*, Maximum likelihood phylogenetic tree of 284 whole RSV-A genomes sampled globally from 2020 to 2024. The circles at branch tips are colored according to sampling location as per the key, with sequences from Africa represented by orange circles, America by pink, Asia by red, Australia by purple, and Europe by green. The 2024 Sydney sequences generated in this study are represented by dark blue circles at branch tips, while those sampled in Sydney in 2022 are indicated by light blue branch tip circles. Nodes with high bootstrap support (> 95%) are annotated by colored diamonds, with black representing 100% bootstrap support, and gray representing 95%–99% bootstrap support. The scale bar represents the nucleotide substitution rate per site. A dashed box highlights the major Sydney clades generated in this study. *B*, Subset maximum likelihood phylogenetic tree illustrating the majority of 2024 Sydney RSV-A sequences generated in this study, rooted by the most recent common ancestor. As per A), colored branch tip circles indicate sampling location. Nodal bootstrap support (%) is illustrated by black (100%), gray (95%–99%), white (80%–95%), or pink (<80%) diamonds. The scale bar represents the expected number of nucleotide substitutions per site.

**Figure 2.**
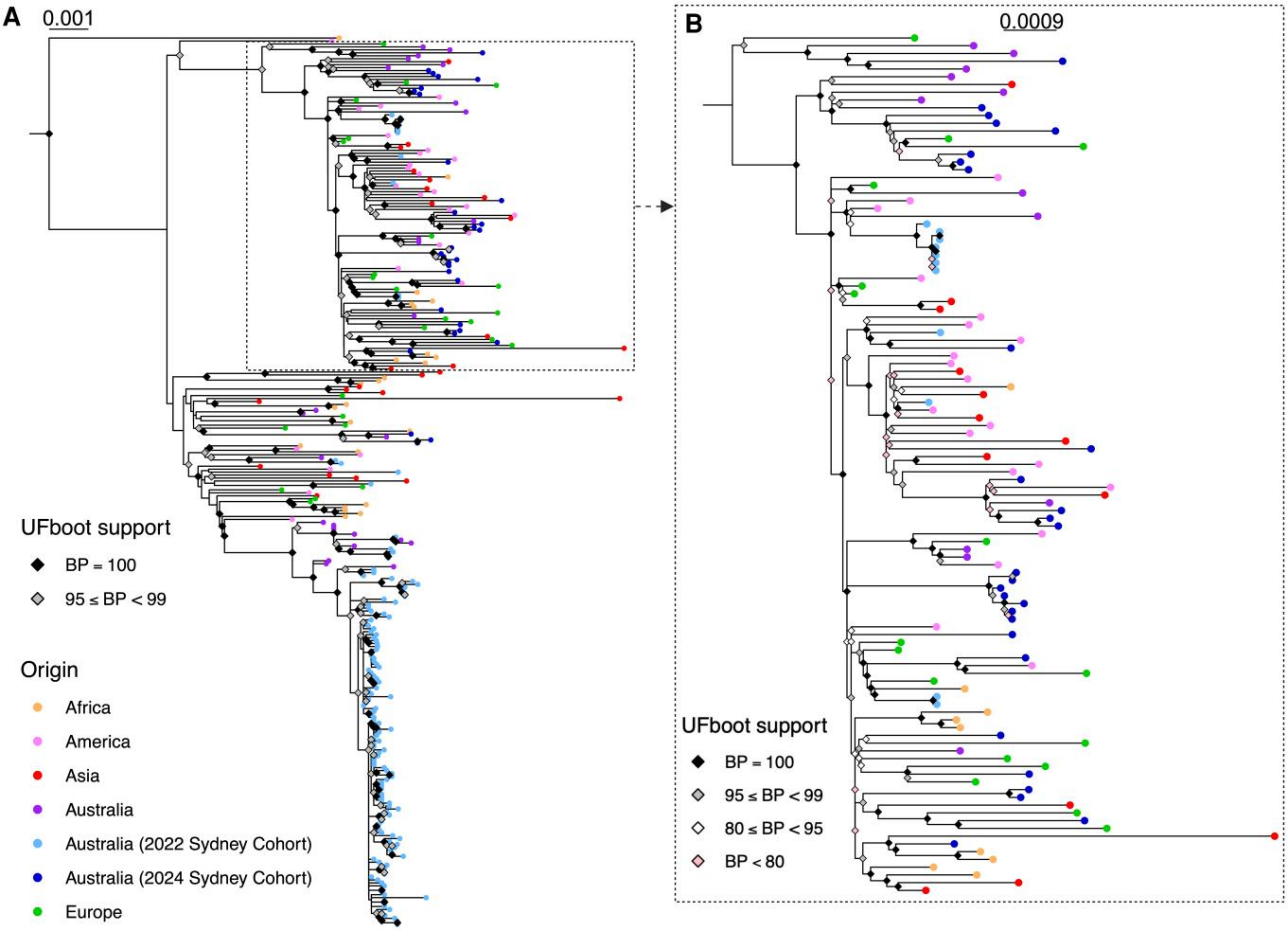
Phylogeny of RSV-B. *A*, Maximum likelihood phylogenetic tree of 297 whole RSV-B genomes sampled globally from 2020 to 2024. Branch tip circles are colored according to sampling location as per the legend included, with sequences from Africa indicated by orange, America by pink, Asia by red, Australia by purple, and Europe by green. The 2024 Sydney sequences generated in this study are represented by dark blue circles at branch tips, while those sampled in Sydney in 2022 are indicated by light blue branch tip circles. Nodes with high bootstrap support (>95%) are annotated with colored diamonds, with black representing 100% bootstrap support and gray representing 95%–99% bootstrap support. The scale bar indicates the nucleotide substitution rate per site. A dashed box highlights the major Sydney clades sampled in this study. *B*, Subset maximum likelihood phylogenetic tree illustrating the majority of Sydney RSV-B sequences generated in this study, rooted by the most recent common ancestor. As per A), branch tip circle colors represent sampling location. Bootstrap support is indicated by a black (100%), gray (95%–99%), white (80%–95%), or pink (<80%) diamond. The scale bar represents the expected number of nucleotide substitutions per site.

The phylogenetic relationships of the 68 RSV-A and 32 RSV-B consensus genomes from this study, 252 (124 RSV-A, 128 RSV-B) global sequences, and 229 (92 RSV-A and 137 RSV-B) 2022 Sydney sequences previously generated by Walker et al [2] were estimated using maximum likelihood analyses. RSV-A sequences generated in the present study were distributed among internationally circulating viruses in the inferred phylogenetic tree rather than forming an independent “Sydney 2024” cluster (Figure 1). There was minimal clustering between the 2024 Sydney sequences and other Australian sequences. In contrast, the majority of 2022 Sydney sequences were placed within a strongly supported (100% bootstrap support) monophyletic group with other Australian sequences and one sequence sampled in Asia. Similar trends in phylogenetic relationships were inferred among RSV-B sequences (Figure 2). The 2024 Sydney RSV-B sequences were placed in different clades of the phylogenetic tree among international sequences, whereas the majority of 2022 Sydney sequences formed a monophyletic cluster with other Australian sequences. In both the RSV-A and RSV-B trees, the long branch lengths between the 2024 and 2022 Sydney RSV sequences indicate a notable genetic distance (Figures 1 and 2).

### Characterization of F Protein Variants

The RSV genomes generated in this study were compared with prototypic references NC_038235.1 (RSV-A) and NC_001781.1 (RSV-B) to identify mutations within the *F* gene. For antigenic site mutations detected in this study, *in vitro* phenotyping data were also collated from existing literature for reference (Table 2). No amino acid substitutions were observed within the nirsevimab or palivizumab-binding sites of RSV-A genomes. All RSV-A samples carried a N276S substitution, adjacent to the palivizumab-binding site (residues 262–275) in antigenic site II, andV152I and L178V substitution within antigenic site V (Table 2) [34]. An additional 4 antigenic site substitutions, I379V (n = 66, 97%) and V384I (n = 57, 84%) in site I and M447V (n = 67, 99%) in site IV were pleomorphic in RSV-A (Table 2).

**Table 2.** Frequency, Relative Frequency (%)^b^, and Predicted Therapeutic Effect^c^ of RSV Fusion Protein Amino Acid Substitutions Within Antigenic Sites in 68 RSV-A and 32 RSV-B Sequences Generated From the Remnant Diagnostic Nasopharyngeal Swabs of 100 Infants < 12 Months Old Who Presented to Sydney Children's Hospital, Randwick, Australia Between March and August, 2024

Subtype	Antigenic Site	Amino Acid Substitution	Frequency Within Cohort, *N* (%)	Fold Change in IC_50_ of Nirsevimab^a^	Fold Change in IC_50_ of Palivizumab
RSV-A	I	I379V	66 (97)	-	-
		V384I	57 (84)	-	-
	II	N276S	68 (100)	-	1.47 [31]
	III	S377I	1 (1)	-	-
		S377N	14 (21)	-	1.02 [31]
	IV	M447V	67 (99)	-	-
		S466N	1 (1)	-	0.96 [31]
	V	V152I	68 (100)	-	-
		L178V	68 (100)	-	-
	VI	G329R	1 (1)	-	-
RSV-B	Ø	K80E	1 (3)	-	-
		Q94R	1 (3)	-	-
		**I206M**	**32 (100)**	**5.0–5.02 [32, 33]**	**1.96–2.0 [32, 33]**
		**Q209R**	**32 (100)**	**0.47–0.5 (32–34)**	**3.1–3.12 (32–34)**
		**S211N**	**32 (100)**	**1.14–1.2 [9, 32, 34]**	**1.9 [32, 34]**
	I	R42K	5 (16)	-	-
		F45L	32 (100)	-	0.4–2.4 [31]
		L381I	2 (6)	-	-
		S389P	26 (81)	-	-
	IV	L467F	1 (3)	-	-
	V	LLS171LQL^d^	32 (100)	0.88–1.12 [32, 34]	1.88–2.3 [32, 34]
		S190N	29 (91)	2.17 [32]	2.62 [32]
		K191R	32 (100)	1.26–1.3 [32–34]	2.7–2.73 [32–34]
	VI	K327N	3 (9)	-	-

^a^Abbreviation: IC50, half-maximal inhibitory concentration.

Fusion protein variants found within the nirsevimab-binding site (residues 62–69 and 196–212) are mentioned in bold [34].

^b^Relative frequency (%) given as a percentage of samples of the same subtype containing a given amino acid substitution.

^c^Therapeutic effect reported as fold-change in the half-maximal inhibitory concentration (IC_50_) of a given therapeutic against mutant viruses compared to reference wild-type viruses in *in vitro* neutralization assays. Of note, no studies to date have examined the effect of fusion protein mutations on neutralization by sera from Abrysvo vaccinated individuals nor infants exposed to maternal antibodies elicited by Abrysvo.

^d^Amino acid substitutions caused by an in-frame altering nucleotide mutation, thus are always co-occurring.

All RSV-B samples carried co-occurring I206M:Q209R:S211N substitutions within the nirsevimab-binding site (antigenic site Ø) (Table 2) [34]. The I206M mutation is known to confer resistance (5-fold increase in IC_50_) to nirsevimab when detected alone but not when detected as a co-occurring mutation as found here [34]. Additionally, all RSV-B samples also harbored an F45L substitution in site I, along with K191R and LLS171LQL substitutions in site V. The S389P (n = 26, 81%) and S190N (n = 29, 91%) substitutions in antigenic sites I and V, respectively, were also common among RSV-B samples (Table 2).

Recently, Liang et al [35] identified 4 regions of the F protein (α-helix 1, α-helix 5, refolding region 1, and refolding region 2) that undergo structural rearrangements during the transition from pre- to post-fusion conformational states. As mutations within these regions can indirectly affect antibody binding, variants outside defined antigenic sites were also included in the analysis (Supplementary Appendix Table 5). All RSV-A samples carried 8 mutations outside of antigenic sites (L4P, A8T, F20L, G25S, P102A, K124N, V139G, and S540A). Three nonantigenic site mutations (L8S, A103V and N234T) were observed in all RSV-B samples (Supplementary Appendix Table 5). The mutability of positions 8 and 103 were shared between subtypes at high frequencies (> 87%) (Supplementary Appendix Table 5). No α-helix 1 or α-helix 5 mutations were identified in RSV-A samples. RSV-B samples carried K80E and Q94R substitutions in α-helix 1 and N234T in α-helix 5 (Table 2 and Supplementary Appendix Table 5). Mutations within refolding region 1 (RR1) and refolding region 2 (RR2) were observed in both subtypes. In RSV-A samples, these included V152I, L178V and V139G substitutions in RR1 and S466N in RR2, whereas in RSV-B samples LLS171LQL, S190N, K191R, I206M, Q209R, and S211N substitutions were found in RR1, and a L467F substitution was observed in RR2 (Table 2 and Supplementary Appendix Table 5).

## DISCUSSION

Over the past 5 years, the circulation and genomic diversity of respiratory viruses have been dramatically impacted by the COVID-19 pandemic and related public health interventions. Several studies, both locally and internationally, reported the collapse of RSV lineage diversity during this period, reflecting the broader disruption of viral transmission networks [7, 8, 36]. In our study, phylogenetic analysis revealed a substantial rebound in RSV genomic diversity in Sydney, Australia, during 2024, compared with sequences similarly sampled in Sydney during a 2022 surge in case numbers [2, 7]. Sequences from these previous seasons formed geographically distinct monophyletic clusters with short branch lengths, indicating little sequence divergence and suggesting sustained local transmission within the context of reduced international travel (Figures 1 and 2). The sequences generated in this study showed no evidence of a predominant Sydney cluster and were instead interspersed among globally circulating viral lineages detected since 2020. These relationships imply that RSV circulating in Sydney during 2024 was derived from multiple independent introductions from overseas, rather than persistence of locally evolving lineages. In contrast to the relatively homogenous A.D.3.1/B.D.E.4 circulation observed in 2022 [2], we observed the emergence of lineages A.D.1.5, A.D.5.1, B.D.E.1.2, B.D.E.1.8, and B.D.E.5, which were first identified in Argentina, the United Kingdom, the USA, Côte d’lvoire, and Kenya, respectively [37]. These lineages had not been circulating widely in Australia prior to 2024 and are reflective of increased lineage turnover and within-season genetic diversity. Notably, these lineages were not detected in Western Australia during the same season [38], which may be due in part to substantial differences in inbound travel patterns and volume. Indeed, state-based clustering of RSV has previously been demonstrated in Australia, suggesting interstate mixing minimally contributes to RSV diversity [39].

Although the RSV F protein is considered highly conserved, an accumulation of amino acid mutations within key antigenic sites has been reported in recent years [34]. Within Sydney-derived RSV-A sequences, the frequency of the N276S substitution in antigenic site II increased from 7% in 2022 to 100% in 2024 [2]. Among RSV-B genomes, the S190N substitution (site V) and the nirsevimab-binding site mutation S211N (site Ø) likewise increased from 7% to 100% in over the same period [2]. Whilst the antigenic mutations observed in this study are not known to confer therapeutic resistance, such mutations have been detected internationally at low frequencies (<1%) in the absence of selective pressure resulting from therapeutic use [34, 40–43]. Nirsevimab is particularly vulnerable to the emergence of escape variants due to its monoclonal design [44]. Suboptimal dosing in infants has been shown to facilitate the selection of resistance-associated substitutions (I64T, K68E, N208S) [32], and a nationwide observational study of children who received nirsevimab in France in 2024 reported resistance-associated mutations at positions 64 and 208 in 8% of RSV-B breakthrough infections [9]. Together, these findings underscore the importance of ongoing genomic surveillance as RSV prophylactic therapeutics are implemented in Australia through the National RSV Mother and Infant Program (commenced February 2025), and as global uptake of monoclonal antibody and vaccine-based interventions continue to expand.

It has recently been shown that mutations outside of defined antigenic sites or in distant regions can alter F protein conformation and antibody binding [35]. For example, the L305I substitution in site III modified the structural arrangement of distant antigenic sites Ø and II, reducing neutralization by both monoclonal and polyclonal antibodies [45]. These findings highlight that residues beyond established antigenic regions—particularly within α-helices and refolding domains—may act as conformational switches influencing epitope accessibility. Thus, many mutations, including those identified in this study (Appendix Supplementary Table 5), remain undefined and warrant phenotypic characterization to determine their potential impact on RSV therapeutic efficacy.

A limitation of our study was that samples were collected from a single pediatric hospital (1 of 2 tertiary children's hospitals in Sydney) during the 2024 season. Thus, our sample selection was limited to infants seeking healthcare and may not be representative of milder RSV cases within the community, or those occurring in other age groups. Although genomes were obtained from a single RSV season, our study built upon our own previous genomic analyses from a 2022 RSV resurgence [2]. This enabled longitudinal comparison across postpandemic seasons, providing temporal context. Future studies may overcome sampling limitations by using complementary approaches such as wastewater-based genomic surveillance to facilitate near real-time resolution of viral lineage abundance in the wider community, as has been performed for SARS-CoV-2 since 2020 [46]. While cost and resource constraints make population-wide genomic surveillance of RSV cases difficult to sustain, wastewater-based surveillance offers a scalable option to fill this gap, analogous to COVID-19 where wastewater monitoring has increasingly replaced broad clinical sequencing for tracking viral diversity and lineage abundance.

This study establishes an important preintervention baseline for RSV genomic diversity in Australian infants. The rebound in genomic diversity following pandemic-related bottlenecks, coupled with the detection of globally distributed lineages, underscores the dynamic nature of RSV transmission and evolution. Continued genomic surveillance will be essential to monitor future viral adaptation under increasing therapeutic selection pressures.

## Supplementary Material

ofag192_Supplementary_Data

## Supplementary Data

Supplementary materials are available at *Open Forum Infectious Diseases* online. Consisting of data provided by the authors to benefit the reader, the posted materials are not copyedited and are the sole responsibility of the authors, so questions or comments should be addressed to the corresponding author.
