# Supplementary material for: Genomic Surveillance of Respiratory Syncytial Virus in Sydney Reveals Rebound in Postpandemic Viral Diversity
Source: Open Forum Infect Dis. 2026 Apr 2;13(4):ofag192. doi: 10.1093/ofid/ofag192 (PMC13097446; doi:10.1093/ofid/ofag192)
Supplement: ofag192_Supplementary_Data [file ofag192_supplementary_data.docx]

**Appendix**

Genomic surveillance of respiratory syncytial virus in Sydney reveals rebound in post-pandemic viral diversity

Julia MS Herkes, Charles SP Foster, Alice Michie, Adam W Bartlett, William D Rawlinson, Gregory J Walker

**This file contains:**

Table S1. Metadata of complete RSV genomes downloaded from GISAID and Sequence Read Archive databases for phylogenetic analysis.

Table S2. Metadata of historical reference genomes.

Table S3. Metadata of sequenced RSV specimens.

Table S4. Sample characteristics.

Table S5. Amino acid substitutions outside of the RSV fusion protein antigenic sites.

Reference List for Appendix.

**Table S1** Metadata of complete RSV-A and RSV-B sequences (> 14,900 nt) sampled from Australia and internationally between 2020 – 2024, including the date of sample collection^†^ as well as the country and region each sample was collected in. Samples were downloaded from the public databases GISAID and NCBI Sequence Read Archive in May, 2025 (*1*)

| Subtype | Accession Number | Date of Collection (dd/mm/yyyy) | Country | Region |
| --- | --- | --- | --- | --- |
| RSV-A | EPI_ISL_1653938 | 22/12/2020 | Australia | Oceania |
| RSV-A | EPI_ISL_2811729 | 26/11/2020 | Australia | Oceania |
| RSV-A | EPI_ISL_2839254 | 03/11/2020 | Australia | Oceania |
| RSV-A | EPI_ISL_2839322 | 24/11/2020 | Australia | Oceania |
| RSV-A | EPI_ISL_2839410 | 18/11/2020 | Australia | Oceania |
| RSV-A | EPI_ISL_15896139 | 23/06/2022 | Australia | Oceania |
| RSV-A | EPI_ISL_15896144 | 15/05/2022 | Australia | Oceania |
| RSV-A | EPI_ISL_15896177 | 13/06/2022 | Australia | Oceania |
| RSV-A | EPI_ISL_17066777 | 19/09/2022 | Australia | Oceania |
| RSV-A | EPI_ISL_19869529 | 31/05/2022 | Australia | Oceania |
| RSV-A | EPI_ISL_19561430 | 24/03/2024 | Australia | Oceania |
| RSV-A | EPI_ISL_19561433 | 14/04/2024 | Australia | Oceania |
| RSV-A | EPI_ISL_19561489 | 24/07/2024 | Australia | Oceania |
| RSV-A | EPI_ISL_19561524 | 07/06/2024 | Australia | Oceania |
| RSV-A | EPI_ISL_19561533 | 22/06/2024 | Australia | Oceania |
| RSV-A | EPI_ISL_19005608 | 22/08/2023 | Australia | Oceania |
| RSV-A | EPI_ISL_19015688 | 22/09/2023 | Australia | Oceania |
| RSV-A | EPI_ISL_19015779 | 13/11/2023 | Australia | Oceania |
| RSV-A | EPI_ISL_19015786 | 16/09/2023 | Australia | Oceania |
| RSV-A | EPI_ISL_19053791 | 26/02/2023 | Australia | Oceania |
| RSV-A | EPI_ISL_11817041 | 05/02/2021 | Australia | Oceania |
| RSV-A | EPI_ISL_18976647 | 04/01/2021 | Australia | Oceania |
| RSV-A | EPI_ISL_19869522 | 18/03/2021 | Australia | Oceania |
| RSV-A | EPI_ISL_2543768 | 05/01/2021 | Australia | Oceania |
| RSV-A | EPI_ISL_2543805 | 07/03/2021 | Australia | Oceania |
| RSV-A | SAMN38194943 | 25/06/2022 | Australia | Oceania |
| RSV-A | SAMN38194950 | 10/07/2022 | Australia | Oceania |
| RSV-A | SAMN38194874 | 03/08/2022 | Australia | Oceania |
| RSV-A | SAMN38194906 | 02/06/2022 | Australia | Oceania |
| RSV-A | SAMN38194896 | 25/05/2022 | Australia | Oceania |
| RSV-A | SAMN38194963 | 22/07/2022 | Australia | Oceania |
| RSV-A | SAMN38194972 | 24/07/2022 | Australia | Oceania |
| RSV-A | SAMN38194974 | 19/07/2022 | Australia | Oceania |
| RSV-A | SAMN38194869 | 08/07/2022 | Australia | Oceania |
| RSV-A | SAMN38194882 | 10/07/2022 | Australia | Oceania |
| RSV-A | SAMN38194927 | 05/07/2022 | Australia | Oceania |
| RSV-A | SAMN38194898 | 02/06/2022 | Australia | Oceania |
| RSV-A | SAMN38194886 | 30/05/2022 | Australia | Oceania |
| RSV-A | SAMN38194961 | 26/07/2022 | Australia | Oceania |
| RSV-A | SAMN38194900 | 26/06/2022 | Australia | Oceania |
| RSV-A | SAMN38194938 | 21/06/2022 | Australia | Oceania |
| RSV-A | SAMN38194934 | 12/06/2022 | Australia | Oceania |
| RSV-A | SAMN38194923 | 05/06/2022 | Australia | Oceania |
| RSV-A | SAMN38194957 | 13/07/2022 | Australia | Oceania |
| RSV-A | SAMN38194954 | 14/07/2022 | Australia | Oceania |
| RSV-A | SAMN38194941 | 19/06/2022 | Australia | Oceania |
| RSV-A | SAMN38194939 | 19/06/2022 | Australia | Oceania |
| RSV-A | SAMN38194978 | 18/06/2022 | Australia | Oceania |
| RSV-A | SAMN38194870 | 08/07/2022 | Australia | Oceania |
| RSV-A | SAMN38194916 | 07/06/2022 | Australia | Oceania |
| RSV-A | SAMN38194893 | 28/05/2022 | Australia | Oceania |
| RSV-A | SAMN38194932 | 11/06/2022 | Australia | Oceania |
| RSV-A | SAMN38194962 | 21/07/2022 | Australia | Oceania |
| RSV-A | SAMN38194965 | 17/07/2022 | Australia | Oceania |
| RSV-A | SAMN38194947 | 26/06/2022 | Australia | Oceania |
| RSV-A | SAMN38194948 | 25/06/2022 | Australia | Oceania |
| RSV-A | SAMN38194976 | 05/08/2022 | Australia | Oceania |
| RSV-A | SAMN38194922 | 05/06/2022 | Australia | Oceania |
| RSV-A | SAMN38194975 | 19/07/2022 | Australia | Oceania |
| RSV-A | SAMN38194918 | 07/06/2022 | Australia | Oceania |
| RSV-A | SAMN38194966 | 02/07/2022 | Australia | Oceania |
| RSV-A | SAMN38194920 | 06/06/2022 | Australia | Oceania |
| RSV-A | SAMN38194945 | 24/06/2022 | Australia | Oceania |
| RSV-A | SAMN38194902 | 21/07/2022 | Australia | Oceania |
| RSV-A | SAMN38194883 | 12/07/2022 | Australia | Oceania |
| RSV-A | SAMN38194911 | 01/06/2022 | Australia | Oceania |
| RSV-A | SAMN38194890 | 29/05/2022 | Australia | Oceania |
| RSV-A | SAMN38194889 | 30/05/2022 | Australia | Oceania |
| RSV-A | SAMN38194891 | 30/05/2022 | Australia | Oceania |
| RSV-A | SAMN38194956 | 10/07/2022 | Australia | Oceania |
| RSV-A | SAMN38194909 | 01/06/2022 | Australia | Oceania |
| RSV-A | SAMN38194946 | 25/06/2022 | Australia | Oceania |
| RSV-A | SAMN38194868 | 09/05/2022 | Australia | Oceania |
| RSV-A | SAMN38194913 | 31/05/2022 | Australia | Oceania |
| RSV-A | SAMN38194917 | 06/06/2022 | Australia | Oceania |
| RSV-A | SAMN38194901 | 17/07/2022 | Australia | Oceania |
| RSV-A | SAMN38194925 | 13/06/2022 | Australia | Oceania |
| RSV-A | SAMN38194931 | 14/06/2022 | Australia | Oceania |
| RSV-A | SAMN38194907 | 31/05/2022 | Australia | Oceania |
| RSV-A | SAMN38194873 | 07/08/2022 | Australia | Oceania |
| RSV-A | SAMN38194903 | 07/07/2022 | Australia | Oceania |
| RSV-A | SAMN38194969 | 02/07/2022 | Australia | Oceania |
| RSV-A | SAMN38194866 | 24/05/2022 | Australia | Oceania |
| RSV-A | SAMN38194897 | 02/06/2022 | Australia | Oceania |
| RSV-A | SAMN38194908 | 02/06/2022 | Australia | Oceania |
| RSV-A | SAMN38194968 | 01/07/2022 | Australia | Oceania |
| RSV-A | SAMN38194929 | 03/07/2022 | Australia | Oceania |
| RSV-A | SAMN38194875 | 04/08/2022 | Australia | Oceania |
| RSV-A | SAMN38194914 | 01/06/2022 | Australia | Oceania |
| RSV-A | SAMN38194899 | 12/06/2022 | Australia | Oceania |
| RSV-A | SAMN38194872 | 30/06/2022 | Australia | Oceania |
| RSV-A | SAMN38194928 | 06/07/2022 | Australia | Oceania |
| RSV-A | SAMN38194915 | 01/06/2022 | Australia | Oceania |
| RSV-A | SAMN38194884 | 22/09/2022 | Australia | Oceania |
| RSV-A | SAMN38194949 | 08/07/2022 | Australia | Oceania |
| RSV-A | SAMN38194930 | 04/07/2022 | Australia | Oceania |
| RSV-A | SAMN38194924 | 14/06/2022 | Australia | Oceania |
| RSV-A | SAMN38194912 | 02/06/2022 | Australia | Oceania |
| RSV-A | SAMN38194958 | 29/07/2022 | Australia | Oceania |
| RSV-A | SAMN38194973 | 23/07/2022 | Australia | Oceania |
| RSV-A | SAMN38194942 | 20/06/2022 | Australia | Oceania |
| RSV-A | SAMN38194952 | 17/07/2022 | Australia | Oceania |
| RSV-A | SAMN38194879 | 27/06/2022 | Australia | Oceania |
| RSV-A | SAMN38194977 | 27/06/2022 | Australia | Oceania |
| RSV-A | SAMN38194887 | 25/05/2022 | Australia | Oceania |
| RSV-A | SAMN38194864 | 15/05/2022 | Australia | Oceania |
| RSV-A | SAMN38194919 | 07/06/2022 | Australia | Oceania |
| RSV-A | SAMN38194953 | 12/07/2022 | Australia | Oceania |
| RSV-A | SAMN38194910 | 03/06/2022 | Australia | Oceania |
| RSV-A | SAMN38194885 | 06/07/2022 | Australia | Oceania |
| RSV-A | SAMN38194940 | 19/06/2022 | Australia | Oceania |
| RSV-A | SAMN38194935 | 13/06/2022 | Australia | Oceania |
| RSV-A | SAMN38194863 | 11/05/2022 | Australia | Oceania |
| RSV-A | SAMN38194892 | 28/05/2022 | Australia | Oceania |
| RSV-A | SAMN38194865 | 16/05/2022 | Australia | Oceania |
| RSV-A | SAMN38194881 | 07/08/2022 | Australia | Oceania |
| RSV-A | SAMN38194937 | 15/06/2022 | Australia | Oceania |
| RSV-A | EPI_ISL_19090990 | 24/01/2022 | South Korea | Asia |
| RSV-A | EPI_ISL_19709942 | 20/09/2022 | Japan | Asia |
| RSV-A | EPI_ISL_19749564 | 01/12/2022 | China | Asia |
| RSV-A | EPI_ISL_19879220 | 06/10/2022 | India | Asia |
| RSV-A | EPI_ISL_19881559 | 22/08/2022 | India | Asia |
| RSV-A | EPI_ISL_19481658 | 20/04/2024 | Qatar | Asia |
| RSV-A | EPI_ISL_19709861 | 07/05/2024 | Japan | Asia |
| RSV-A | EPI_ISL_19723109 | 11/09/2024 | Laos | Asia |
| RSV-A | EPI_ISL_19783076 | 03/09/2024 | Cambodia | Asia |
| RSV-A | EPI_ISL_19091004 | 06/03/2023 | South Korea | Asia |
| RSV-A | EPI_ISL_19147773 | 14/08/2023 | Japan | Asia |
| RSV-A | EPI_ISL_19147784 | 31/08/2023 | Japan | Asia |
| RSV-A | EPI_ISL_19719118 | 13/09/2023 | Laos | Asia |
| RSV-A | EPI_ISL_19723094 | 20/10/2023 | Laos | Asia |
| RSV-A | EPI_ISL_18851816 | 15/10/2021 | Kuwait | Asia |
| RSV-A | EPI_ISL_19090984 | 20/12/2021 | South Korea | Asia |
| RSV-A | EPI_ISL_19709895 | 30/08/2021 | Japan | Asia |
| RSV-A | EPI_ISL_19814929 | 10/02/2021 | China | Asia |
| RSV-A | EPI_ISL_19814947 | 15/12/2021 | China | Asia |
| RSV-A | EPI_ISL_12970426 | 22/01/2020 | Philippines | Asia |
| RSV-A | EPI_ISL_12970428 | 09/03/2020 | Philippines | Asia |
| RSV-A | EPI_ISL_18482729 | 11/11/2020 | China | Asia |
| RSV-A | EPI_ISL_18482744 | 11/02/2020 | China | Asia |
| RSV-A | EPI_ISL_18482784 | 13/01/2020 | China | Asia |
| RSV-A | EPI_ISL_16289039 | 16/11/2022 | Spain | Europe |
| RSV-A | EPI_ISL_17995770 | 20/10/2022 | Germany | Europe |
| RSV-A | EPI_ISL_18321013 | 28/06/2022 | Ireland | Europe |
| RSV-A | EPI_ISL_18789629 | 04/10/2022 | France | Europe |
| RSV-A | EPI_ISL_19131457 | 31/10/2022 | Spain | Europe |
| RSV-A | EPI_ISL_18880305 | 05/01/2024 | Spain | Europe |
| RSV-A | EPI_ISL_19046536 | 26/02/2024 | Italy | Europe |
| RSV-A | EPI_ISL_19174365 | 30/01/2024 | Spain | Europe |
| RSV-A | EPI_ISL_19630263 | 08/02/2024 | France | Europe |
| RSV-A | EPI_ISL_19630744 | 04/01/2024 | France | Europe |
| RSV-A | EPI_ISL_18329462 | 03/04/2023 | Italy | Europe |
| RSV-A | EPI_ISL_19131601 | 12/01/2023 | Spain | Europe |
| RSV-A | EPI_ISL_19174342 | 10/10/2023 | Spain | Europe |
| RSV-A | EPI_ISL_19544815 | 10/2023 | Scotland | Europe |
| RSV-A | EPI_ISL_19630544 | 27/10/2023 | France | Europe |
| RSV-A | EPI_ISL_18272380 | 09/2021 | Scotland | Europe |
| RSV-A | EPI_ISL_18682164 | 25/06/2021 | Spain | Europe |
| RSV-A | EPI_ISL_18789184 | 21/02/2021 | France | Europe |
| RSV-A | EPI_ISL_18789226 | 14/03/2021 | France | Europe |
| RSV-A | EPI_ISL_19442407 | 22/09/2021 | Scotland | Europe |
| RSV-A | EPI_ISL_15753559 | 03/01/2020 | United Kingdom | Europe |
| RSV-A | EPI_ISL_17995682 | 03/02/2020 | Germany | Europe |
| RSV-A | EPI_ISL_18682249 | 08/01/2020 | Spain | Europe |
| RSV-A | EPI_ISL_18789113 | 07/01/2020 | France | Europe |
| RSV-A | EPI_ISL_19063386 | 14/02/2020 | Italy | Europe |
| RSV-A | EPI_ISL_11055721 | 31/01/2022 | South Africa | Africa |
| RSV-A | EPI_ISL_11055728 | 17/02/2022 | South Africa | Africa |
| RSV-A | EPI_ISL_11055730 | 16/02/2022 | South Africa | Africa |
| RSV-A | EPI_ISL_17368044 | 14/04/2022 | South Africa | Africa |
| RSV-A | EPI_ISL_18240645 | 22/05/2022 | Senegal | Africa |
| RSV-A | EPI_ISL_19220694 | 23/04/2024 | South Africa | Africa |
| RSV-A | EPI_ISL_19465888 | 10/07/2024 | South Africa | Africa |
| RSV-A | EPI_ISL_19465891 | 23/05/2024 | South Africa | Africa |
| RSV-A | EPI_ISL_19510845 | 19/04/2024 | South Africa | Africa |
| RSV-A | EPI_ISL_19510851 | 31/05/2024 | South Africa | Africa |
| RSV-A | EPI_ISL_17559320 | 02/03/2023 | South Africa | Africa |
| RSV-A | EPI_ISL_17559369 | 01/02/2023 | South Africa | Africa |
| RSV-A | EPI_ISL_18933039 | 24/11/2023 | Senegal | Africa |
| RSV-A | EPI_ISL_19621812 | 11/10/2023 | South Africa | Africa |
| RSV-A | EPI_ISL_19621889 | 27/06/2023 | South Africa | Africa |
| RSV-A | EPI_ISL_11055735 | 27/01/2021 | South Africa | Africa |
| RSV-A | EPI_ISL_12529643 | 25/03/2021 | South Africa | Africa |
| RSV-A | EPI_ISL_12529648 | 08/04/2021 | South Africa | Africa |
| RSV-A | EPI_ISL_14039047 | 06/2021 | Uganda | Africa |
| RSV-A | EPI_ISL_19544316 | 28/10/2021 | Tunisia | Africa |
| RSV-A | EPI_ISL_17308728 | 17/04/2020 | Mozambique | Africa |
| RSV-A | EPI_ISL_18061464 | 20/01/2020 | South Africa | Africa |
| RSV-A | EPI_ISL_19049264 | 18/03/2020 | South Africa | Africa |
| RSV-A | EPI_ISL_19752983 | 18/09/2020 | South Africa | Africa |
| RSV-A | EPI_ISL_6208726 | 08/01/2020 | Egypt | Africa |
| RSV-A | EPI_ISL_16681408 | 31/10/2022 | USA^*^ | Americas |
| RSV-A | EPI_ISL_18939421 | 10/2022 | USA | Americas |
| RSV-A | EPI_ISL_19137622 | 10/2022 | USA | Americas |
| RSV-A | EPI_ISL_19423146 | 20/10/2022 | Panama | Americas |
| RSV-A | EPI_ISL_19576078 | 08/11/2022 | USA | Americas |
| RSV-A | EPI_ISL_19140044 | 22/01/2024 | USA | Americas |
| RSV-A | EPI_ISL_19216023 | 22/03/2024 | USA | Americas |
| RSV-A | EPI_ISL_19230594 | 09/01/2024 | USA | Americas |
| RSV-A | EPI_ISL_19230607 | 03/02/2024 | USA | Americas |
| RSV-A | EPI_ISL_19774945 | 03/09/2024 | USA | Americas |
| RSV-A | EPI_ISL_17950205 | 17/01/2023 | USA | Americas |
| RSV-A | EPI_ISL_17950234 | 24/01/2023 | USA | Americas |
| RSV-A | EPI_ISL_18742333 | 24/10/2023 | USA | Americas |
| RSV-A | EPI_ISL_19159510 | 06/11/2023 | USA | Americas |
| RSV-A | EPI_ISL_19568438 | 13/10/2023 | Panama | Americas |
| RSV-A | EPI_ISL_18939395 | 12/2021 | USA | Americas |
| RSV-A | EPI_ISL_19006995 | 21/09/2021 | USA | Americas |
| RSV-A | EPI_ISL_19125841 | 17/06/2021 | USA | Americas |
| RSV-A | EPI_ISL_19442392 | 21/06/2021 | USA | Americas |
| RSV-A | EPI_ISL_19575998 | 13/10/2021 | USA | Americas |
| RSV-A | EPI_ISL_17089184 | 03/2020 | USA | Americas |
| RSV-A | EPI_ISL_18143556 | 04/01/2020 | USA | Americas |
| RSV-A | EPI_ISL_19048052 | 08/01/2020 | Canada | Americas |
| RSV-A | EPI_ISL_19048924 | 26/02/2020 | Canada | Americas |
| RSV-A | EPI_ISL_19423119 | 08/01/2020 | Panama | Americas |
| RSV-B | EPI_ISL_11817067 | 11/03/2021 | Australia | Oceania |
| RSV-B | EPI_ISL_11817068 | 13/03/2021 | Australia | Oceania |
| RSV-B | EPI_ISL_11817085 | 09/04/2021 | Australia | Oceania |
| RSV-B | EPI_ISL_11817093 | 26/03/2021 | Australia | Oceania |
| RSV-B | EPI_ISL_11817098 | 04/04/2021 | Australia | Oceania |
| RSV-B | EPI_ISL_11817110 | 10/12/2020 | Australia | Oceania |
| RSV-B | EPI_ISL_11817115 | 15/12/2020 | Australia | Oceania |
| RSV-B | EPI_ISL_11817117 | 17/12/2020 | Australia | Oceania |
| RSV-B | EPI_ISL_11817122 | 22/12/2020 | Australia | Oceania |
| RSV-B | EPI_ISL_11817129 | 31/12/2020 | Australia | Oceania |
| RSV-B | EPI_ISL_16714811 | 15/10/2022 | Australia | Oceania |
| RSV-B | EPI_ISL_17066832 | 05/12/2022 | Australia | Oceania |
| RSV-B | EPI_ISL_18657035 | 23/06/2023 | Australia | Oceania |
| RSV-B | EPI_ISL_18976623 | 12/09/2022 | Australia | Oceania |
| RSV-B | EPI_ISL_19005631 | 26/08/2023 | Australia | Oceania |
| RSV-B | EPI_ISL_19005679 | 06/08/2023 | Australia | Oceania |
| RSV-B | EPI_ISL_19005709 | 31/05/2023 | Australia | Oceania |
| RSV-B | EPI_ISL_19561341 | 29/02/2024 | Australia | Oceania |
| RSV-B | EPI_ISL_19561349 | 04/03/2024 | Australia | Oceania |
| RSV-B | EPI_ISL_19561444 | 05/05/2024 | Australia | Oceania |
| RSV-B | EPI_ISL_19561452 | 18/05/2024 | Australia | Oceania |
| RSV-B | EPI_ISL_19561479 | 04/07/2024 | Australia | Oceania |
| RSV-B | EPI_ISL_19869662 | 18/08/2022 | Australia | Oceania |
| RSV-B | EPI_ISL_19869679 | 07/11/2022 | Australia | Oceania |
| RSV-B | EPI_ISL_19869794 | 18/07/2023 | Australia | Oceania |
| RSV-B | EPI_ISL_19869833 | 29/08/2023 | Australia | Oceania |
| RSV-B | EPI_ISL_2156818 | 22/02/2020 | Australia | Oceania |
| RSV-B | SAMN38195059 | 02/06/2022 | Australia | Oceania |
| RSV-B | SAMN38195108 | 10/07/2022 | Australia | Oceania |
| RSV-B | SAMN38195100 | 30/07/2022 | Australia | Oceania |
| RSV-B | SAMN38195110 | 16/07/2022 | Australia | Oceania |
| RSV-B | SAMN38195042 | 25/05/2022 | Australia | Oceania |
| RSV-B | SAMN38194983 | 17/05/2022 | Australia | Oceania |
| RSV-B | SAMN38195016 | 05/06/2022 | Australia | Oceania |
| RSV-B | SAMN38195070 | 06/06/2022 | Australia | Oceania |
| RSV-B | SAMN38195101 | 11/07/2022 | Australia | Oceania |
| RSV-B | SAMN38195116 | 12/07/2022 | Australia | Oceania |
| RSV-B | SAMN38195132 | 25/07/2022 | Australia | Oceania |
| RSV-B | SAMN38195143 | 20/07/2022 | Australia | Oceania |
| RSV-B | SAMN38194984 | 21/05/2022 | Australia | Oceania |
| RSV-B | SAMN38195061 | 03/06/2022 | Australia | Oceania |
| RSV-B | SAMN38195152 | 23/09/2022 | Australia | Oceania |
| RSV-B | SAMN38195036 | 26/05/2022 | Australia | Oceania |
| RSV-B | SAMN38195129 | 26/07/2022 | Australia | Oceania |
| RSV-B | SAMN38195149 | 23/07/2022 | Australia | Oceania |
| RSV-B | SAMN38195046 | 31/05/2022 | Australia | Oceania |
| RSV-B | SAMN38195028 | 07/07/2022 | Australia | Oceania |
| RSV-B | SAMN38195017 | 26/06/2022 | Australia | Oceania |
| RSV-B | SAMN38195069 | 06/06/2022 | Australia | Oceania |
| RSV-B | SAMN38194991 | 05/08/2022 | Australia | Oceania |
| RSV-B | SAMN38195018 | 20/06/2022 | Australia | Oceania |
| RSV-B | SAMN38195081 | 14/06/2022 | Australia | Oceania |
| RSV-B | SAMN38195060 | 02/06/2022 | Australia | Oceania |
| RSV-B | SAMN38195004 | 04/08/2022 | Australia | Oceania |
| RSV-B | SAMN38195085 | 20/06/2022 | Australia | Oceania |
| RSV-B | SAMN38195150 | 21/07/2022 | Australia | Oceania |
| RSV-B | SAMN38194995 | 29/06/2022 | Australia | Oceania |
| RSV-B | SAMN38194998 | 05/08/2022 | Australia | Oceania |
| RSV-B | SAMN38195148 | 23/07/2022 | Australia | Oceania |
| RSV-B | SAMN38195034 | 30/05/2022 | Australia | Oceania |
| RSV-B | SAMN38195124 | 30/07/2022 | Australia | Oceania |
| RSV-B | SAMN38195147 | 21/07/2022 | Australia | Oceania |
| RSV-B | SAMN38195002 | 05/08/2022 | Australia | Oceania |
| RSV-B | SAMN38195067 | 07/06/2022 | Australia | Oceania |
| RSV-B | SAMN38195026 | 05/07/2022 | Australia | Oceania |
| RSV-B | SAMN38195134 | 28/07/2022 | Australia | Oceania |
| RSV-B | SAMN38195131 | 25/07/2022 | Australia | Oceania |
| RSV-B | SAMN38195005 | 21/09/2022 | Australia | Oceania |
| RSV-B | SAMN38195065 | 31/05/2022 | Australia | Oceania |
| RSV-B | SAMN38195000 | 09/09/2022 | Australia | Oceania |
| RSV-B | SAMN38195062 | 01/06/2022 | Australia | Oceania |
| RSV-B | SAMN38195156 | 09/06/2022 | Australia | Oceania |
| RSV-B | SAMN38195071 | 06/06/2022 | Australia | Oceania |
| RSV-B | SAMN38195140 | 22/07/2022 | Australia | Oceania |
| RSV-B | SAMN38195035 | 30/05/2022 | Australia | Oceania |
| RSV-B | SAMN38195037 | 27/05/2022 | Australia | Oceania |
| RSV-B | SAMN38195027 | 08/07/2022 | Australia | Oceania |
| RSV-B | SAMN38195144 | 19/07/2022 | Australia | Oceania |
| RSV-B | SAMN38195044 | 29/05/2022 | Australia | Oceania |
| RSV-B | SAMN38195074 | 04/06/2022 | Australia | Oceania |
| RSV-B | SAMN38195051 | 29/05/2022 | Australia | Oceania |
| RSV-B | SAMN38195133 | 28/07/2022 | Australia | Oceania |
| RSV-B | SAMN38195113 | 14/07/2022 | Australia | Oceania |
| RSV-B | SAMN38195121 | 03/08/2022 | Australia | Oceania |
| RSV-B | SAMN38195153 | 29/07/2022 | Australia | Oceania |
| RSV-B | SAMN38195057 | 28/05/2022 | Australia | Oceania |
| RSV-B | SAMN38195139 | 17/07/2022 | Australia | Oceania |
| RSV-B | SAMN38195117 | 11/07/2022 | Australia | Oceania |
| RSV-B | SAMN38195146 | 24/07/2022 | Australia | Oceania |
| RSV-B | SAMN38195119 | 09/07/2022 | Australia | Oceania |
| RSV-B | SAMN38195106 | 11/07/2022 | Australia | Oceania |
| RSV-B | SAMN38195054 | 31/05/2022 | Australia | Oceania |
| RSV-B | SAMN38195092 | 02/07/2022 | Australia | Oceania |
| RSV-B | SAMN38195118 | 08/07/2022 | Australia | Oceania |
| RSV-B | SAMN38195072 | 06/06/2022 | Australia | Oceania |
| RSV-B | SAMN38195038 | 28/05/2022 | Australia | Oceania |
| RSV-B | SAMN38195055 | 30/05/2022 | Australia | Oceania |
| RSV-B | SAMN38194988 | 09/07/2022 | Australia | Oceania |
| RSV-B | SAMN38195009 | 12/06/2022 | Australia | Oceania |
| RSV-B | SAMN38195087 | 26/06/2022 | Australia | Oceania |
| RSV-B | SAMN38195091 | 03/07/2022 | Australia | Oceania |
| RSV-B | SAMN38195073 | 04/06/2022 | Australia | Oceania |
| RSV-B | SAMN38194985 | 17/05/2022 | Australia | Oceania |
| RSV-B | SAMN38195020 | 01/06/2022 | Australia | Oceania |
| RSV-B | SAMN38195021 | 09/06/2022 | Australia | Oceania |
| RSV-B | SAMN38195158 | 12/06/2022 | Australia | Oceania |
| RSV-B | SAMN38195086 | 20/06/2022 | Australia | Oceania |
| RSV-B | SAMN38195010 | 10/07/2022 | Australia | Oceania |
| RSV-B | SAMN38194999 | 05/07/2022 | Australia | Oceania |
| RSV-B | SAMN38195137 | 26/07/2022 | Australia | Oceania |
| RSV-B | SAMN38195030 | 27/05/2022 | Australia | Oceania |
| RSV-B | SAMN38195138 | 29/07/2022 | Australia | Oceania |
| RSV-B | SAMN38195024 | 12/07/2022 | Australia | Oceania |
| RSV-B | SAMN38195076 | 13/06/2022 | Australia | Oceania |
| RSV-B | SAMN38195001 | 04/08/2022 | Australia | Oceania |
| RSV-B | SAMN38195115 | 13/07/2022 | Australia | Oceania |
| RSV-B | SAMN38195097 | 28/07/2022 | Australia | Oceania |
| RSV-B | SAMN38195107 | 09/07/2022 | Australia | Oceania |
| RSV-B | SAMN38195078 | 15/06/2022 | Australia | Oceania |
| RSV-B | SAMN38195098 | 29/07/2022 | Australia | Oceania |
| RSV-B | SAMN38195126 | 30/07/2022 | Australia | Oceania |
| RSV-B | SAMN38194986 | 27/05/2022 | Australia | Oceania |
| RSV-B | SAMN38195039 | 27/05/2022 | Australia | Oceania |
| RSV-B | SAMN38195096 | 28/07/2022 | Australia | Oceania |
| RSV-B | SAMN38194992 | 03/07/2022 | Australia | Oceania |
| RSV-B | SAMN38195064 | 01/06/2022 | Australia | Oceania |
| RSV-B | SAMN38195090 | 03/07/2022 | Australia | Oceania |
| RSV-B | SAMN38195040 | 29/05/2022 | Australia | Oceania |
| RSV-B | SAMN38195015 | 18/07/2022 | Australia | Oceania |
| RSV-B | SAMN38195031 | 22/05/2022 | Australia | Oceania |
| RSV-B | SAMN38195120 | 11/07/2022 | Australia | Oceania |
| RSV-B | SAMN38195007 | 06/09/2022 | Australia | Oceania |
| RSV-B | SAMN38195029 | 27/05/2022 | Australia | Oceania |
| RSV-B | SAMN38194981 | 15/05/2022 | Australia | Oceania |
| RSV-B | SAMN38195112 | 15/07/2022 | Australia | Oceania |
| RSV-B | SAMN38194994 | 01/07/2022 | Australia | Oceania |
| RSV-B | SAMN38195050 | 25/05/2022 | Australia | Oceania |
| RSV-B | SAMN38195122 | 31/07/2022 | Australia | Oceania |
| RSV-B | SAMN38195063 | 31/05/2022 | Australia | Oceania |
| RSV-B | SAMN38195022 | 02/07/2022 | Australia | Oceania |
| RSV-B | SAMN38195080 | 14/06/2022 | Australia | Oceania |
| RSV-B | SAMN38195145 | 23/07/2022 | Australia | Oceania |
| RSV-B | SAMN38195011 | 03/07/2022 | Australia | Oceania |
| RSV-B | SAMN38195014 | 05/06/2022 | Australia | Oceania |
| RSV-B | SAMN38195093 | 07/07/2022 | Australia | Oceania |
| RSV-B | SAMN38195068 | 06/06/2022 | Australia | Oceania |
| RSV-B | SAMN38195045 | 30/05/2022 | Australia | Oceania |
| RSV-B | SAMN38195049 | 30/05/2022 | Australia | Oceania |
| RSV-B | SAMN38194993 | 02/07/2022 | Australia | Oceania |
| RSV-B | SAMN38195154 | 26/07/2022 | Australia | Oceania |
| RSV-B | SAMN38195075 | 03/06/2022 | Australia | Oceania |
| RSV-B | SAMN38195052 | 31/05/2022 | Australia | Oceania |
| RSV-B | SAMN38195151 | 19/07/2022 | Australia | Oceania |
| RSV-B | SAMN38195127 | 26/07/2022 | Australia | Oceania |
| RSV-B | SAMN38195025 | 04/07/2022 | Australia | Oceania |
| RSV-B | SAMN38194987 | 22/05/2022 | Australia | Oceania |
| RSV-B | SAMN38195125 | 03/08/2022 | Australia | Oceania |
| RSV-B | SAMN38195012 | 20/06/2022 | Australia | Oceania |
| RSV-B | SAMN38195135 | 12/07/2022 | Australia | Oceania |
| RSV-B | SAMN38195089 | 01/07/2022 | Australia | Oceania |
| RSV-B | SAMN38195079 | 14/06/2022 | Australia | Oceania |
| RSV-B | SAMN38195123 | 30/07/2022 | Australia | Oceania |
| RSV-B | SAMN38195047 | 30/05/2022 | Australia | Oceania |
| RSV-B | SAMN38195109 | 10/07/2022 | Australia | Oceania |
| RSV-B | EPI_ISL_18042527 | 13/07/2022 | Philippines | Asia |
| RSV-B | EPI_ISL_18042540 | 02/07/2022 | Philippines | Asia |
| RSV-B | EPI_ISL_18231081 | 16/10/2022 | Bangladesh | Asia |
| RSV-B | EPI_ISL_18279071 | 12/07/2023 | Cambodia | Asia |
| RSV-B | EPI_ISL_18482716 | 03/01/2020 | China | Asia |
| RSV-B | EPI_ISL_18482755 | 30/10/2020 | China | Asia |
| RSV-B | EPI_ISL_18845918 | 15/08/2022 | Kuwait | Asia |
| RSV-B | EPI_ISL_19090964 | 15/03/2023 | South Korea | Asia |
| RSV-B | EPI_ISL_19696224 | 14/09/2023 | Philippines | Asia |
| RSV-B | EPI_ISL_19709901 | 04/11/2020 | Japan | Asia |
| RSV-B | EPI_ISL_19709904 | 26/11/2021 | Japan | Asia |
| RSV-B | EPI_ISL_19709946 | 17/06/2021 | Japan | Asia |
| RSV-B | EPI_ISL_19709956 | 19/06/2023 | Japan | Asia |
| RSV-B | EPI_ISL_19739195 | 04/03/2024 | Laos | Asia |
| RSV-B | EPI_ISL_19739197 | 03/05/2024 | Laos | Asia |
| RSV-B | EPI_ISL_19749471 | 13/01/2020 | China | Asia |
| RSV-B | EPI_ISL_19749481 | 20/01/2021 | China | Asia |
| RSV-B | EPI_ISL_19783116 | 27/09/2024 | Cambodia | Asia |
| RSV-B | EPI_ISL_19783141 | 11/10/2024 | Cambodia | Asia |
| RSV-B | EPI_ISL_19814988 | 22/12/2021 | China | Asia |
| RSV-B | EPI_ISL_19826471 | 05/02/2020 | Japan | Asia |
| RSV-B | EPI_ISL_19863366 | 19/08/2024 | Bangladesh | Asia |
| RSV-B | EPI_ISL_19879214 | 17/09/2022 | India | Asia |
| RSV-B | EPI_ISL_19879255 | 18/02/2023 | India | Asia |
| RSV-B | EPI_ISL_6314642 | 02/04/2021 | China | Asia |
| RSV-B | EPI_ISL_14084081 | 03/01/2022 | Spain | Europe |
| RSV-B | EPI_ISL_15753134 | 23/01/2020 | Spain | Europe |
| RSV-B | EPI_ISL_1647581 | 06/01/2020 | England | Europe |
| RSV-B | EPI_ISL_1647588 | 03/03/2020 | England | Europe |
| RSV-B | EPI_ISL_16714580 | 07/04/2022 | England | Europe |
| RSV-B | EPI_ISL_16714613 | 07/07/2021 | England | Europe |
| RSV-B | EPI_ISL_16714717 | 25/07/2022 | England | Europe |
| RSV-B | EPI_ISL_18005809 | 17/03/2020 | Germany | Europe |
| RSV-B | EPI_ISL_18277121 | 08/2021 | Scotland | Europe |
| RSV-B | EPI_ISL_18329481 | 06/01/2023 | Italy | Europe |
| RSV-B | EPI_ISL_18537509 | 27/10/2023 | France | Europe |
| RSV-B | EPI_ISL_18569162 | 29/09/2023 | England | Europe |
| RSV-B | EPI_ISL_18648349 | 09/11/2023 | Spain | Europe |
| RSV-B | EPI_ISL_18682123 | 10/03/2022 | Spain | Europe |
| RSV-B | EPI_ISL_18682140 | 18/05/2021 | Spain | Europe |
| RSV-B | EPI_ISL_18682144 | 30/05/2021 | Spain | Europe |
| RSV-B | EPI_ISL_18789294 | 11/05/2021 | France | Europe |
| RSV-B | EPI_ISL_19049190 | 13/01/2020 | Russia | Europe |
| RSV-B | EPI_ISL_19276917 | 12/09/2022 | England | Europe |
| RSV-B | EPI_ISL_19446462 | 29/10/2023 | England | Europe |
| RSV-B | EPI_ISL_19629718 | 18/11/2024 | France | Europe |
| RSV-B | EPI_ISL_19656535 | 20/11/2024 | Spain | Europe |
| RSV-B | EPI_ISL_19718039 | 31/12/2024 | Spain | Europe |
| RSV-B | EPI_ISL_19725135 | 14/12/2024 | Ireland | Europe |
| RSV-B | EPI_ISL_19744839 | 28/12/2024 | France | Europe |
| RSV-B | EPI_ISL_11055785 | 05/02/2021 | South Africa | Africa |
| RSV-B | EPI_ISL_11055790 | 18/03/2021 | South Africa | Africa |
| RSV-B | EPI_ISL_11055797 | 08/06/2021 | South Africa | Africa |
| RSV-B | EPI_ISL_11055803 | 28/06/2021 | South Africa | Africa |
| RSV-B | EPI_ISL_11055804 | 07/07/2021 | South Africa | Africa |
| RSV-B | EPI_ISL_14769859 | 20/05/2022 | South Africa | Africa |
| RSV-B | EPI_ISL_15728619 | 01/07/2022 | South Africa | Africa |
| RSV-B | EPI_ISL_17998374 | 03/05/2022 | South Africa | Africa |
| RSV-B | EPI_ISL_18228536 | 10/06/2022 | Senegal | Africa |
| RSV-B | EPI_ISL_19049093 | 17/03/2020 | South Africa | Africa |
| RSV-B | EPI_ISL_19220734 | 23/05/2023 | South Africa | Africa |
| RSV-B | EPI_ISL_19220770 | 19/02/2024 | South Africa | Africa |
| RSV-B | EPI_ISL_19220837 | 05/07/2023 | South Africa | Africa |
| RSV-B | EPI_ISL_19220857 | 08/04/2024 | South Africa | Africa |
| RSV-B | EPI_ISL_19220864 | 09/04/2024 | South Africa | Africa |
| RSV-B | EPI_ISL_19220873 | 24/04/2024 | South Africa | Africa |
| RSV-B | EPI_ISL_19510764 | 14/05/2024 | South Africa | Africa |
| RSV-B | EPI_ISL_19714723 | 15/02/2020 | Madagascar | Africa |
| RSV-B | EPI_ISL_19714764 | 26/02/2020 | Madagascar | Africa |
| RSV-B | EPI_ISL_19714775 | 25/11/2020 | Madagascar | Africa |
| RSV-B | EPI_ISL_19754361 | 15/05/2020 | South Africa | Africa |
| RSV-B | EPI_ISL_19754399 | 13/04/2023 | South Africa | Africa |
| RSV-B | EPI_ISL_19754410 | 04/04/2023 | South Africa | Africa |
| RSV-B | EPI_ISL_19754420 | 23/03/2023 | South Africa | Africa |
| RSV-B | EPI_ISL_19754424 | 07/03/2022 | South Africa | Africa |
| RSV-B | EPI_ISL_16132420 | 11/2022 | USA | Americas |
| RSV-B | EPI_ISL_16681327 | 07/07/2022 | USA | Americas |
| RSV-B | EPI_ISL_18061174 | 04/02/2020 | Canada | Americas |
| RSV-B | EPI_ISL_18143466 | 31/08/2021 | USA | Americas |
| RSV-B | EPI_ISL_18143475 | 07/09/2021 | USA | Americas |
| RSV-B | EPI_ISL_18143524 | 04/01/2022 | USA | Americas |
| RSV-B | EPI_ISL_18694983 | 29/09/2021 | USA | Americas |
| RSV-B | EPI_ISL_18694985 | 27/03/2023 | USA | Americas |
| RSV-B | EPI_ISL_18695000 | 14/04/2023 | USA | Americas |
| RSV-B | EPI_ISL_18695078 | 16/10/2022 | USA | Americas |
| RSV-B | EPI_ISL_18939594 | 13/01/2023 | USA | Americas |
| RSV-B | EPI_ISL_19006978 | 19/08/2021 | USA | Americas |
| RSV-B | EPI_ISL_19048664 | 06/02/2020 | Mexico | Americas |
| RSV-B | EPI_ISL_19048828 | 25/02/2020 | Mexico | Americas |
| RSV-B | EPI_ISL_19087030 | 13/02/2023 | USA | Americas |
| RSV-B | EPI_ISL_19125710 | 22/02/2020 | USA | Americas |
| RSV-B | EPI_ISL_19125711 | 06/02/2020 | USA | Americas |
| RSV-B | EPI_ISL_19125787 | 10/03/2022 | USA | Americas |
| RSV-B | EPI_ISL_19277538 | 23/09/2023 | USA | Americas |
| RSV-B | EPI_ISL_19575950 | 05/10/2021 | USA | Americas |
| RSV-B | EPI_ISL_19623594 | 21/05/2024 | USA | Americas |
| RSV-B | EPI_ISL_19654783 | 12/08/2024 | USA | Americas |
| RSV-B | EPI_ISL_19654820 | 06/12/2024 | USA | Americas |
| RSV-B | EPI_ISL_19664666 | 26/11/2024 | USA | Americas |
| RSV-B | EPI_ISL_19817437 | 27/12/2024 | Mexico | Americas |
| RSV-B | EPI_ISL_19871981 | 30/12/2024 | USA | Americas |

^*^USA, United States of America.

^†^Complete date of collection was not recorded on GISAID for seven of the randomly selected RSV-A sequences and two of the randomly selected RSV-B sequences.

**Table S2** Metadata of historic, whole RSV genomes sourced from the GISAID and NCBI Nucleotide databases (*1, 2*)

| Subtype | Accession Number | Year of Collection |
| --- | --- | --- |
| RSV-A | EPI_ISL_15771600 | 1956 |
|  | EPI_ISL_15771601 | 1994 |
|  | EPI_ISL_15771603 | 1995 |
|  | EPI_ISL_15771604 | 1995 |
|  | EPI_ISL_15771615 | 1993 |
| RSV-B | MG642025 | 1982 |
|  | MG642027 | 1991 |
|  | MG642037 | 1980 |
|  | MG642043 | 1982 |
|  | MG642045 | 1979 |

**Table S3** Metadata of the 100 consensus RSV genomes generated in this study using a tiled-amplicon based whole-genome sequencing approach from remnant diagnostic nasopharyngeal swabs of RSV-positive infants < 12 months of age who presented to Sydney Children’s Hospital, Randwick, Australia between March – August 2024

| Sequence Name | SRA Accession Number | | Subtype* | Lineage^†^ | Date of Collection (dd/mm/yyyy) | RT-qPCR Ct Value* | Coverage (%) | Mean depth |
| --- | --- | --- | --- | --- | --- | --- | --- | --- |
| 2024RSV-1 | | SAMN53129481 | RSV-A | A.D.1.4 | 30/03/2024 | 22.00 | 98.48 | 818.689 |
| 2024RSV-2 | | SAMN53129482 | RSV-A | A.D.1.4 | 31/03/2024 | 17.15 | 98.63 | 1411.386 |
| 2024RSV-3 | | SAMN53129483 | RSV-A | A.D.1.5 | 03/03/2024 | 21.00 | 98.56 | 1180.864 |
| 2024RSV-4 | | SAMN53129484 | RSV-A | A.D.1.4 | 29/03/2024 | 24.00 | 98.48 | 677.979 |
| 2024RSV-5 | | SAMN53129485 | RSV-A | A.D.5.2 | 29/03/2024 | 26.00 | 94.19 | 867.2 |
| 2024RSV-6 | | SAMN53129486 | RSV-A | A.D.1.5 | 30/03/2024 | 22.00 | 98.35 | 636.221 |
| 2024RSV-7 | | SAMN53129487 | RSV-A | A.D.1.4 | 31/03/2024 | 18.00 | 98.62 | 732.097 |
| 2024RSV-8 | | SAMN53129488 | RSV-A | A.D.1.4 | 25/03/2024 | 24.00 | 98.62 | 742.936 |
| 2024RSV-9 | | SAMN53129489 | RSV-B | B.D.E.1 | 27/03/2024 | 26.00 | 97.12 | 1124.927 |
| 2024RSV-10 | | SAMN53129490 | RSV-A | A.D.1.4 | 28/03/2024 | 22.00 | 98.63 | 1181.88 |
| 2024RSV-11 | | SAMN53129491 | RSV-B | B.D.E.1 | 29/03/2024 | 21.00 | 99.45 | 1205.699 |
| 2024RSV-12 | | SAMN53129492 | RSV-B | B.D.E.1 | 11/03/2024 | 19.00 | 99.45 | 1964.835 |
| 2024RSV-13 | | SAMN53129493 | RSV-B | B.D.E.1 | 12/03/2024 | 28.00 | 97.69 | 1222.602 |
| 2024RSV-14 | | SAMN53129494 | RSV-B | B.D.E.1.2 | 01/03/2024 | 25.00 | 99.45 | 1783.384 |
| 2024RSV-15 | | SAMN53129495 | RSV-B | B.D.E.1 | 28/03/2024 | 19.00 | 99.45 | 1770.363 |
| 2024RSV-16 | | SAMN53129496 | RSV-B | B.D.E.1.2 | 03/03/2024 | 17.00 | 99.45 | 916.581 |
| 2024RSV-17 | | SAMN53129497 | RSV-B | B.D.E.1 | 31/03/2024 | 18.09 | 99.45 | 1348.968 |
| 2024RSV-18 | | SAMN53129498 | RSV-A | A.D.1.4 | 07/03/2024 | 19.00 | 98.63 | 1262.34 |
| 2024RSV-19 | | SAMN53129499 | RSV-B | B.D.E.1 | 21/03/2024 | 20.00 | 99.45 | 1219.125 |
| 2024RSV-20 | | SAMN53129500 | RSV-B | B.D.E.1 | 27/03/2024 | 19.00 | 99.45 | 1680.57 |
| 2024RSV-21 | | SAMN53129501 | RSV-A | A.D.1.4 | 09/04/2024 | 19.79 | 98.61 | 717.129 |
| 2024RSV-22 | | SAMN53129502 | RSV-A | A.D.1.4 | 02/04/2024 | 20.00 | 98.63 | 1513.04 |
| 2024RSV-23 | | SAMN53129503 | RSV-A | A.D.5.2 | 02/04/2024 | 22.00 | 98.35 | 1008.461 |
| 2024RSV-24 | | SAMN53129504 | RSV-A | A.D.3 | 07/04/2024 | 20.53 | 98.46 | 1532.446 |
| 2024RSV-25 | | SAMN53129505 | RSV-A | A.D.1.4 | 14/04/2024 | 16.32 | 98.63 | 1465.471 |
| 2024RSV-26 | | SAMN53129506 | RSV-A | A.D.5.2 | 23/04/2024 | 17.77 | 98.35 | 1123.105 |
| 2024RSV-27 | | SAMN53129507 | RSV-A | A.D.1.4 | 19/04/2024 | 17.27 | 98.57 | 1294.784 |
| 2024RSV-28 | | SAMN53129508 | RSV-A | A.D.1.4 | 25/04/2024 | 17.03 | 98.63 | 1705.453 |
| 2024RSV-29 | | SAMN53129509 | RSV-A | A.D.1.4 | 21/04/2024 | 18.93 | 98.63 | 1267.685 |
| 2024RSV-30 | | SAMN53129510 | RSV-A | A.D.1.4 | 29/04/2024 | 16.57 | 98.63 | 809.263 |
| 2024RSV-31 | | SAMN53129511 | RSV-B | B.D.E.1 | 07/04/2024 | 19.77 | 99.45 | 1731.959 |
| 2024RSV-32 | | SAMN53129512 | RSV-B | B.D.E.1 | 10/04/2024 | 25.54 | 97.99 | 1629.543 |
| 2024RSV-33 | | SAMN53129513 | RSV-B | B.D.E.1.2 | 07/04/2024 | 20.36 | 99.45 | 1588.99 |
| 2024RSV-34 | | SAMN53129514 | RSV-B | B.D.E.1 | 07/04/2024 | 27.14 | 98.94 | 1175.685 |
| 2024RSV-35 | | SAMN53129515 | RSV-B | B.D.E.1 | 01/04/2024 | 20.76 | 99.45 | 1584.553 |
| 2024RSV-36 | | SAMN53129516 | RSV-B | B.D.E.1 | 08/04/2024 | 29.24 | 99.34 | 1055.5 |
| 2024RSV-37 | | SAMN53129517 | RSV-B | B.D.E.1 | 08/04/2024 | 18.89 | 99.45 | 555.14 |
| 2024RSV-38 | | SAMN53129518 | RSV-B | B.D.E.1 | 07/04/2024 | 19.70 | 99.45 | 933.821 |
| 2024RSV-39 | | SAMN53129519 | RSV-B | B.D.E.1 | 29/04/2024 | 18.43 | 99.45 | 1437.323 |
| 2024RSV-40 | | SAMN53129520 | RSV-B | B.D.4.1.1 | 29/04/2024 | 17.97 | 99.45 | 1819.923 |
| 2024RSV-41 | | SAMN53129521 | RSV-A | A.D.1.4 | 01/05/2024 | 22.00 | 98.27 | 603.506 |
| 2024RSV-42 | | SAMN53129522 | RSV-B | B.D.E.5 | 10/05/2024 | 19.61 | 99.45 | 1753.765 |
| 2024RSV-43 | | SAMN53129523 | RSV-A | A.D.1.4 | 12/05/2024 | 19.51 | 98.53 | 706.326 |
| 2024RSV-44 | | SAMN53129524 | RSV-A | A.D.1.4 | 10/05/2024 | 25.03 | 97.44 | 797.87 |
| 2024RSV-45 | | SAMN53129525 | RSV-A | A.D.1.4 | 11/05/2024 | 23.95 | 98.63 | 825.731 |
| 2024RSV-46 | | SAMN53129526 | RSV-B | B.D.E.1.8 | 26/05/2024 | 15.11 | 99.45 | 1649.865 |
| 2024RSV-47 | | SAMN53129527 | RSV-A | A.D.1.4 | 24/05/2024 | 21.25 | 98.62 | 853 |
| 2024RSV-48 | | SAMN53129528 | RSV-A | A.D.3 | 14/05/2024 | 19.08 | 98.35 | 788.152 |
| 2024RSV-49 | | SAMN53129529 | RSV-B | B.D.E.1 | 20/05/2024 | 16.90 | 99.45 | 1441.408 |
| 2024RSV-50 | | SAMN53129530 | RSV-B | B.D.E.5 | 11/05/2024 | 19.03 | 98.15 | 525.308 |
| 2024RSV-51 | | SAMN53129531 | RSV-B | B.D.E.1.2 | 17/05/2024 | 16.19 | 99.45 | 826.793 |
| 2024RSV-52 | | SAMN53129532 | RSV-A | A.D.1.4 | 20/05/2024 | 19.89 | 97.67 | 518.917 |
| 2024RSV-53 | | SAMN53129533 | RSV-A | A.D.5.2 | 19/05/2024 | 22.10 | 98.12 | 884.316 |
| 2024RSV-54 | | SAMN53129534 | RSV-B | B.D.E.1.2 | 13/05/2024 | 19.62 | 99.95 | 1611.113 |
| 2024RSV-55 | | SAMN53129535 | RSV-B | B.D.E.1 | 16/05/2024 | 19.15 | 99.45 | 1378.247 |
| 2024RSV-56 | | SAMN53129536 | RSV-A | A.D.1.4 | 27/05/2024 | 24.18 | 96.96 | 646 |
| 2024RSV-57 | | SAMN53129537 | RSV-B | B.D.E.1 | 16/05/2024 | 22.07 | 99.45 | 1150.112 |
| 2024RSV-58 | | SAMN53129538 | RSV-B | B.D.E.1.2 | 24/05/2024 | 21.25 | 98.41 | 831.626 |
| 2024RSV-59 | | SAMN53129539 | RSV-A | A.D.1.5 | 18/05/2024 | 20.97 | 97.96 | 522.734 |
| 2024RSV-60 | | SAMN53129540 | RSV-B | B.D.E.1 | 01/05/2024 | 18.00 | 99.45 | 1138.407 |
| 2024RSV-61 | | SAMN53129541 | RSV-A | A.D.1.4 | 29/06/2024 | 18.00 | 98.00 | 828.277 |
| 2024RSV-62 | | SAMN53129542 | RSV-A | A.D.3 | 30/06/2024 | 21.00 | 94.99 | 637.176 |
| 2024RSV-63 | | SAMN53129543 | RSV-A | A.D.1.4 | 29/06/2024 | 25.00 | 97.35 | 829.287 |
| 2024RSV-64 | | SAMN53129544 | RSV-A | A.D.1.5 | 25/06/2024 | 26.00 | 92.61 | 302.739 |
| 2024RSV-65 | | SAMN53129545 | RSV-A | A.D.1.4 | 27/06/2024 | 20.00 | 98.61 | 686.463 |
| 2024RSV-66 | | SAMN53129546 | RSV-A | A.D.1.5 | 26/06/2024 | 26.00 | 98.34 | 526.678 |
| 2024RSV-67 | | SAMN53129547 | RSV-A | A.D.1.4 | 11/07/2024 | 18.55 | 97.39 | 656.261 |
| 2024RSV-68 | | SAMN53129548 | RSV-A | A.D.1.4 | 19/07/2024 | 21.00 | 96.97 | 581.283 |
| 2024RSV-69 | | SAMN53129549 | RSV-A | A.D.1.5 | 09/07/2024 | 20.83 | 96.53 | 406.521 |
| 2024RSV-70 | | SAMN53129550 | RSV-A | A.D.1.4 | 09/07/2024 | 18.00 | 91.85 | 377.759 |
| 2024RSV-71 | | SAMN53129551 | RSV-A | A.D.5.2 | 18/07/2024 | 21.15 | 97.48 | 414.505 |
| 2024RSV-72 | | SAMN53129552 | RSV-A | A.D.1.4 | 06/07/2024 | 17.00 | 98.63 | 915.04 |
| 2024RSV-73 | | SAMN53129553 | RSV-A | A.D.3 | 19/07/2024 | 19.00 | 96.72 | 925.95 |
| 2024RSV-74 | | SAMN53129554 | RSV-A | A.D.1.4 | 31/07/2024 | 16.00 | 98.61 | 771.758 |
| 2024RSV-75 | | SAMN53129555 | RSV-B | B.D.E.5 | 07/08/2024 | 19.79 | 98.15 | 1296.96 |
| 2024RSV-76 | | SAMN53129556 | RSV-B | B.D.E.1 | 30/07/2024 | 25.00 | 99.10 | 1164.435 |
| 2024RSV-77 | | SAMN53129557 | RSV-A | A.D.1.4 | 23/07/2024 | 19.95 | 98.63 | 888.89 |
| 2024RSV-78 | | SAMN53129558 | RSV-A | A.D.1.5 | 27/07/2024 | 25.00 | 98.35 | 671.489 |
| 2024RSV-79 | | SAMN53129559 | RSV-A | A.D.1.5 | 29/07/2024 | 23.00 | 98.35 | 837.929 |
| 2024RSV-80 | | SAMN53129560 | RSV-A | A.D.1.5 | 23/07/2024 | 21.57 | 98.35 | 795.429 |
| 2024RSV-81 | | SAMN53129561 | RSV-A | A.D.1.4 | 30/07/2024 | 18.00 | 98.00 | 913.223 |
| 2024RSV-82 | | SAMN53129562 | RSV-A | A.D.1.5 | 29/07/2024 | 21.00 | 98.35 | 1073.99 |
| 2024RSV-83 | | SAMN53129563 | RSV-A | A.D.1.5 | 24/07/2024 | 31.00 | 91.92 | 773.556 |
| 2024RSV-84 | | SAMN53129564 | RSV-A | A.D.1.4 | 18/07/2024 | 20.11 | 97.02 | 960.576 |
| 2024RSV-85 | | SAMN53129565 | RSV-A | A.D.1.5 | 07/07/2024 | 28.00 | 96.76 | 884.358 |
| 2024RSV-86 | | SAMN53129566 | RSV-A | A.D.5.2 | 21/08/2024 | 20.00 | 98.34 | 872.92 |
| 2024RSV-87 | | SAMN53129567 | RSV-A | A.D.1.5 | 08/08/2024 | 30.32 | 98.35 | 868.273 |
| 2024RSV-88 | | SAMN53129568 | RSV-A | A.D.1.4 | 25/08/2024 | 17.68 | 98.63 | 1045.22 |
| 2024RSV-89 | | SAMN53129569 | RSV-A | A.D.1.4 | 23/08/2024 | 18.28 | 98.62 | 855.424 |
| 2024RSV-90 | | SAMN53129570 | RSV-A | A.D.5.1 | 05/08/2024 | 19.00 | 92.35 | 683.708 |
| 2024RSV-91 | | SAMN53129571 | RSV-A | A.D.1.5 | 23/08/2024 | 24.78 | 98.35 | 630.928 |
| 2024RSV-92 | | SAMN53129572 | RSV-A | A.D.1.6 | 22/08/2024 | 23.00 | 98.35 | 707.126 |
| 2024RSV-93 | | SAMN53129573 | RSV-A | A.D.5.1 | 04/08/2024 | 23.00 | 94.70 | 923.021 |
| 2024RSV-94 | | SAMN53129574 | RSV-A | A.D.5.2 | 26/08/2024 | 23.00 | 98.35 | 909.344 |
| 2024RSV-95 | | SAMN53129575 | RSV-A | A.D.5.1 | 15/08/2024 | 22.00 | 95.23 | 482.859 |
| 2024RSV-96 | | SAMN53129576 | RSV-A | A.D.3 | 31/08/2024 | 25.85 | 95.11 | 595.357 |
| 2024RSV-97 | | SAMN53129577 | RSV-A | A.D.1.5 | 06/08/2024 | 23.58 | 98.35 | 881.691 |
| 2024RSV-98 | | SAMN53129578 | RSV-A | A.D.5.2 | 29/08/2024 | 22.00 | 98.35 | 957.098 |
| 2024RSV-99 | | SAMN53129579 | RSV-A | A.D.1.5 | 18/08/2024 | 25.00 | 98.35 | 857.391 |
| 2024RSV-100 | | SAMN53129580 | RSV-A | A.D.1.4 | 02/08/2024 | 31.00 | 87.22 | 429.046 |

^*^RSV subtype and Reverse transcription-quantitative polymerase chain reaction cycle threshold (RT-qPCR Ct) value were determined at the time of RSV diagnosis by the Serology and Virology Division, New South Wales Health Pathology using the Allplex Respiratory Panel 1 (Seegene, South Korea) protocol.

^†^Lineages were assigned using nextclade (v3.17.0) based on alignment of consensus genomes to the nextclade dataset 2025-09-09--12-13-13Z (*3*).

**Table S4** Characteristics of RSV-positive remnant clinical nasopharyngeal swabs sampled from 100 infants < 12 months of age who presented to Sydney Children’s Hospital, Randwick, Australia, between March – August 2024

| Characteristics | No. (n= 100) |
| --- | --- |
| RSV Subtype^†^, No. |  |
| RSV-A | 68 |
| RSV-B | 32 |
| RT-qPCR Ct value, median [IQR]^*,‡^ | 20.76 [18.97 – 23.98] |
| Collection month, No. |  |
| March | 20 |
| April | 20 |
| May | 20 |
| June^§^ | 6 |
| July | 19 |
| August | 15 |

^*^Abbreviations: RT-qPCR Ct, reverse transcription-quantitative polymerase chain reaction cycle threshold, IQR, interquartile range.

^†^RSV subtype was determined at the time of diagnosis by the Serology and Virology Division, New South Wales Health Pathology, as per the Allplex Respiratory Panel 1 (Seegene, South Korea) protocol.

^‡^RT-qPCR Ct values were not recorded for thirteen samples (6 RSV-A, 7 RSV-B) collected in May, 2024.

^§^A total of six archived samples collected during June, 2024 were eligible for inclusion in this study.

**Table S5** Frequency and relative frequency (%)^†^ of fusion (F) protein amino acid substitutions observed outside of antigenic sites in 68 RSV-A and 32 RSV-B genomes generated from 100 infants < 12 months who presented to Sydney Children’s Hospital, Randwick, Australia, between March – August, 2024

| Subtype | F protein region^*^ | Amino acid substitution | No. (%) |
| --- | --- | --- | --- |
| RSV-A | SP | E2D | 1 (1) |
|  |  | L3S | 34 (50) |
|  |  | L4P | 68 (100) |
|  |  | A8T | 68 (100) |
|  |  | T12I | 5 (7) |
|  |  | L15F | 1 (1) |
|  |  | T16A | 58 (85) |
|  |  | F20L | 68 (100) |
|  |  | G25S | 68 (100) |
|  | F2 | P101T | 1 (1) |
|  |  | P102A | 68 (100) |
|  |  | T103A | 58 (85) |
|  |  | T103E | 1 (1) |
|  |  | T103V | 1 (1) |
|  |  | N105S | 65 (96) |
|  | P27 | N120Y | 1 (1) |
|  |  | A122T | 57 (84) |
|  |  | K124N | 68 (100) |
|  |  | V127I | 16 (24) |
|  |  | K132I | 4 (6) |
|  |  | R135K | 1 (1) |
|  | F1 | V139G^*^ | 68 (100) |
|  |  | E356D | 1 (1) |
|  | TD | S540A | 68 (100) |
| RSV-B | SP | L8S | 32 (100) |
|  |  | F12I | 7 (22) |
|  |  | L22F | 1 (3) |
|  | F2 | A103V | 32 (100) |
|  | P27 | M115V | 1 (3) |
|  |  | L125P | 1 (3) |
|  | F1 | N234T^*^ | 32 (100) |
|  |  | I402V | 7 (22) |
|  | TD | T529A | 29 (91) |
|  |  | T529V | 3 (9) |
|  |  | S540T | 1 (3) |
|  | CD | K551N | 1 (3) |

^*^Mutations within refolding region 1 (residues 137 – 215) and α-helix 5 (residues 216 – 240) are highlighted in green and pink, respectively (*4*). Abbreviations: SP, signal peptide, TD, transmembrane domain, CD, cytoplasmic domain.

^†^Relative frequency (%) given as the percentage of samples with a given mutation out of the total number of samples of the same subtype.

**References**

1. Elbe S, Buckland-Merrett G. Data, disease and diplomacy: GISAID's innovative contribution to global health. Global Challenges. 2017 Jan 10;1(1):33-46.

2. Sayers EW, Beck J, Bolton EE, Brister JR, Chan J, Connor R, et al. Database resources of the National Center for Biotechnology Information in 2025. Nucleic Acids Res. 2025 Jan 6;53(D1):D20-d9.

3. Aksamentov I, Roemer C, Hodcroft E, Neher R. Nextclade: clade assignment, mutation calling and quality control for viral genomes. Journal of Open Source Softw. 2021 Nov 30;6(67).

4. Liang Y, Shao S, Li XY, Zhao ZX, Liu N, Liu ZM, et al. Mutating a flexible region of the RSV F protein can stabilize the prefusion conformation. Science. 2024 Sep 27;385(6716):1484-91.
